# CRISPR/Cas9 facilitates investigation of neural circuit disease using human iPSCs: mechanism of epilepsy caused by an *SCN1A* loss-of-function mutation

**DOI:** 10.1038/tp.2015.203

**Published:** 2016-01-05

**Authors:** J Liu, C Gao, W Chen, W Ma, X Li, Y Shi, H Zhang, L Zhang, Y Long, H Xu, X Guo, S Deng, X Yan, D Yu, G Pan, Y Chen, L Lai, W Liao, Z Li

**Affiliations:** 1 School of Life Sciences, University of Science and Technology of China, Hefei, China; 2 Key Laboratory of Regenerative Biology, Guangdong Provincial Key Laboratory of Stem Cell and Regenerative Medicine, South China Institute for Stem Cell Biology and Regenerative Medicine, South China Institute for Stem Cell Biology and Regenerative Medicine, Guangzhou Institutes of Biomedicine and Health, Chinese Academy of Sciences, Guangzhou, China; 3 Key Laboratory of Neurogenetics and Channelopathies of Guangdong Province and the Ministry of Education of China, Collaborative Innovation Center for Neurogenetics and Channelopathies, Institute of Neuroscience, The Second Affiliated Hospital of Guangzhou Medical University, Guangzhou, China; 4 Department of Anatomy and Neurobiology, School of Basic Medical Sciences, Central South University, Changsha, China

## Abstract

Mutations in *SCN1A*, the gene encoding the α subunit of Nav1.1 channel, can cause epilepsies with wide ranges of clinical phenotypes, which are associated with the contrasting effects of channel loss-of-function or gain-of-function. In this project, CRISPR/Cas9- and TALEN-mediated genome-editing techniques were applied to induced pluripotent stem cell (iPSC)-based-disease model to explore the mechanism of epilepsy caused by *SCN1A* loss-of-function mutation. By fluorescently labeling GABAergic subtype in iPSC-derived neurons using CRISPR/Cas9, we for the first time performed electrophysiological studies on *SCN1A*-expressing neural subtype and monitored the postsynaptic activity of both inhibitory and excitatory types. We found that the mutation c.A5768G, which led to no current of Nav1.1 in exogenously transfected system, influenced the properties of not only Nav current amount, but also Nav activation in Nav1.1-expressing GABAergic neurons. The two alterations in Nav further reduced the amplitudes and enhanced the thresholds of action potential in patient-derived GABAergic neurons, and led to weakened spontaneous inhibitory postsynaptic currents (sIPSCs) in the patient-derived neuronal network. Although the spontaneous excitatory postsynaptic currents (sEPSCs) did not change significantly, when the frequencies of both sIPSCs and sEPSCs were further analyzed, we found the whole postsynaptic activity transferred from the inhibition-dominated state to excitation in patient-derived neuronal networks, suggesting that changes in sIPSCs alone were sufficient to significantly reverse the excitatory level of spontaneous postsynaptic activity. In summary, our findings fill the gap of our knowledge regarding the relationship between *SCN1A* mutation effect recorded on exogenously transfected cells and on Nav1.1-expressing neurons, and reveal the physiological basis underlying epileptogenesis caused by *SCN1A* loss-of-function mutation.

## Introduction

Mutations in *SCN1A*, the gene that encodes the α subunit of voltage-gated sodium channel Nav1.1, can cause epilepsies with wide ranges of clinical phenotypes, from the relatively benign genetic epilepsy with febrile seizures plus to the more severe Dravet syndrome.^1, 2, 3, 4^ Some of these epilepsies, such as Dravet syndrome,^5^ are therapy resistant, and the treatment of the rest is relied mainly on antiepileptic drugs. There are now more than 20 antiepileptic drugs available in the market that have different action mechanisms and targets.^6^ With limited information regarding the mechanism of epilepsies caused by *SCN1A*, it has been challenging for physicians to choose among drugs that synergistically control seizures while minimizing adverse effects.

The epileptic *SCN1A* mutants heterologously examined in the transfection system show diverse electrophysiological alterations that can be mainly divided into gain-of-function and loss-of-function. Loss-of-function mutations are more common and are associated with more severe clinical manifestations of epilepsy.^7, 8, 9, 10, 11, 12^ Animal models and patient-induced pluripotent stem cell (iPSC)-derived models^13, 14^ exist for many types of the epilepsies. Nonetheless, these studies have failed to yield conclusive insight into the mechanisms for any type of the *SCN1A*-related epilepsies.^15^ Those animal models mainly use Nav1.1 deletion to mimic loss-of-function mutation. Constitutional knockout of Nav1.1 in the mouse brain led to the decrease in Nav current and excitability in GABAergic neurons in the cerebellum and hippocampus, but no detectable alteration in excitatory pyramidal hippocampal neurons.^16, 17^ Later, selective deletion of Nav1.1 in GABAergic neurons was found to be sufficient to cause epilepsy in mouse, and additional deletion of Nav1.1 in excitatory neurons could alleviate the existing epileptic symptom.^18^ These results generated from Nav1.1-deletion animal models indicated that functional impairment of GABAergic neurons might be involved in this disease. However, findings from patient iPSC-derived model showed an increased sodium current density in putative inhibitory and excitatory neurons, in association with increased evoked action potentials (AP) and spontaneous bursting.^19^ The understanding for these apparently paradoxical findings is difficult; especially when the iPSC-derived models used have some inherent limitations. One of the most severe problems of the system provided by iPSCs is that it cannot balance the contradiction that a neural system of both excitatory and inhibitory subtypes is required and that the model should focus specifically on the Nav1.1-expressing neuronal subtype to determine the direct effects of mutation and to identify disease origin.

Epileptic seizure, as a result of failure in balance between neural inhibition and excitation, involves two neural subtypes and the interaction between them through synaptic connection. In the brain, Nav1.1 is expressed predominantly in GABAergic neurons^20^ as well as in a small subpopulation of excitatory pyramidal neurons.^18, 21, 22, 23^ This biased distribution of Nav1.1 suggests that the disease originates from a certain subtype and the two subtypes participate in different ways during the disease process. Clearly, the existing iPSC-derived model cannot provide a system containing both types of neurons, and meanwhile allowing for distinguishing the different subtypes during electrophysiological study and focusing on the Nav1.1-expressing subtype. In addition, the iPSC-derived model is based on comparisons between different genetic backgrounds, yet the genetic background was reported to be able to modulate disease severity of epilepsy,^24, 25^ which may further interfere with the understanding of the observed disease-related difference.

Hence, by combining the recently rapidly evolving genome-editing techniques^26, 27, 28^ with iPSC-derived model, we corrected the disease-causing mutation in epilepsy patient iPSCs to serve as an isogenic control (corrected) in addition to the allogenetic control iPSCs (control) from a healthy individual. In addition, the tdTomato gene was introduced into the genome of these iPSCs to label the GABAergic neurons in the differentiated neuronal networks that contained both GABAergic and glutamatergic neurons. Using this system, we investigated the epilepsy caused by *SCN1A* loss-of-function mutation on the Nav1.1-expressing neuronal subtype as well as the whole neuronal network.

## Materials and methods

### Description of epilepsy patient

In brief, the patient is a 10-year-old boy and was diagnosed with partial epilepsy with antecedent febrile seizures (PEFS+), presented with febrile seizures at the age of 6 months and afebrile seizures at the age of 20 months. The electroencephalogram mainly showed 4–6-Hz eight-rhythm background, accompanied by irregular spike and wave complex and polyspike and wave complex. Seizure types were unilateral clonic, complex partial seizures and secondarily generalized tonic-clonic seizures. His father was a mosaic carrier with ∼25% mutation and was diagnosed with febrile seizures. His affected half-sister carried the same mutation and was also diagnosed with PEFS+ however, there were minor differences between their symptoms. More description about this patient can be found in the report by Shi *et al.*^29^

The *SCN1A* mutation carried by the patient was NM_001165963.1:c.5768A>G (Q1923R), which caused the substitution of Q to R in the conserved C-terminal of the Nav1.1 α subunit. This missense mutation was discovered by DNA sequencing and denaturing high-performance liquid chromatography, and was identified as heterozygous.^29^

### hiPSC derivation and maintenance

The patient iPSCs were acquired by reprogramming the fibroblasts as described in previous work.^13^ The control iPSCs were reprogrammed from the fibroblasts of a healthy female fetus of 16 weeks using the same method. For the following experiments, all iPSCs were cultured on Matrigel (BD Biosciences, Franklin, NJ, USA) in mTeSR1 (Stemcell Technologies, Vancouver, BC, Canada).

### CRISPR/Cas9-mediated knock-in and TALEN-mediated gene correction

The guide RNA primer was designed as shown in Supplementary Table 1 and was cloned into the plasmid U6-gRNA-BbsI. The tdTomato complementary DNA was cloned upstream of a loxP-flanked PGK-puromycin cassette between two homology arms. For targeting, 1 × 10^6^ iPSCs were electroporated with 10 μg of donor DNA, 5 μg of Cas9 DNA and 1 μg of U6-gRNA-BbsI DNA using a Human Stem Cell Nucleofector Kit 2 (Lonza, Basel, Switzerland) and A23 procedure of the Amaxa Nucleofector 11 Device (Lonza). Single positive clones were selected by puromycin (0.5 μg ml^−1^). The selected clones were verified with genomic PCR using KOD-Plus-Neo (ToYoBo, Osaka, Japan) according to the manufacturer's instructions. TALEN-mediated gene correction was conducted as we described before.^30^

### Electrophysiology

Electrophysiological recordings were performed at room temperature using a whole-cell, voltage- or current-clamp technique. Whole-cell recordings were made with 6−9-MΩ borosilicate glass electrodes. Specific protocols were depicted in each figure.

For AP and voltage-gated channel recordings, the external solution used was artificial cerebrospinal fluid containing (in mM): NaCl (127), KCI (3), MgSO_4_ (1) NaHCO_3_ (26), NaH_2_PO_4_ (1.25), CaCl_2_ (2) and D-glucose (10), pH 7.3–7.4. The internal solution contained (in mM): potassium-methylsulfonate (140), NaCl (5), CaCl_2_ (1), HEPES (10), EGTA (0.2), ATPNa_2_ (3) and GTPNa_2_ (0.4). AP was recorded at resting membrane potential (RMP). The AP threshold was taken as the potential where the rate of rise crossed 6.5 V s^−1^. AP properties were analyzed using Clampfit 10.2 (Molecular Devices, Sunnyvale, CA, USA).

To record the characteristics of voltage-dependent sodium channels on neurons, 0.1 mM CdCl_2_ was added to artificial cerebrospinal fluid to block calcium channels. For recordings of transfected cells, we used the internal and external solutions that were described before.^31^ Series resistance was compensated 87–95%. Sodium current properties were analyzed using Clampfit 10.2. The activation curves for the sodium channel were obtained by fitting the data using the Boltzmann equation, *I*/*I*_max_=1−{1+exp[(*V*_m_−*V*_1/2_)/*k*]}^−1^, where *I* is the current amplitude activated by a given step of voltage pulse, *I*_max_ is the maximum response of the given voltage pulse, *V*_m_ is the voltage pulse and *V*_1/2_ is the voltage pulse eliciting a half-maximal response.

Spontaneous postsynaptic activity was assessed referring to methods described previously.^32^ The spontaneous inhibitory postsynaptic currents (sIPSCs) were recorded at the reversal potential of ionotropic glutamate receptors (0 mV); moreover, the spontaneous excitatory postsynaptic currents (sEPSCs) were recorded at the reversal potential of GABA receptors (−65 mV). Both were recorded in the presence of 1 μM strychnine, which was bath applied. Postsynaptic events were detected by MiniAnalysis and verified manually. The ratio of sIPSC frequency to sEPSC frequency for each neuron was defined as the quotient of each neuron's sIPSC frequency and the average sEPSC frequency of the sample.

### Statistical analyses

No statistical methods were used to predetermine sample sizes; however, our sample sizes were similar to those described in previous publications.^16, 32, 33^ Exact sample size for each group was given in the specific figure legends. The data from neuronal recordings were collected with the investigators blind to the genotypes of the cell lines. The animal used in the teratoma formation assay was picked randomly. There were no multiple comparisons used. Average values are expressed as the mean±s.e.m. Data were statistically compared by unpaired one-tailed *t*-test using Origin 7.5 (OriginLab, Northampton, MA, USA). *P<*0.05 was considered significant.

See Supplementary Materials and Methods.

## Results

### Generation of patient iPSCs and gene correction by TALEN

The iPSCs of patient were acquired by retroviral transduction of Oct4, Sox2, Klf4 and c-Myc into patient fibroblasts. The obtained iPSCs exhibited typical human ES cell morphology and showed the characteristics of pluripotency (Supplementary Figures 1a–e).

**Figure 1:**
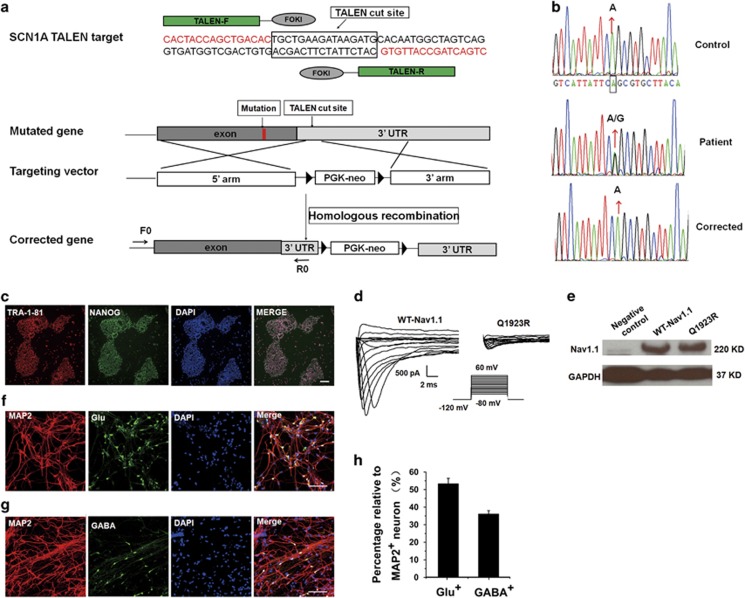
Mutation correction in patient iPSCs by TALEN and neural differentiation. (**a**) Scheme of TALEN-mediated homologous recombination. (**b**) Sequencing maps of PCR product from genome DNA by primers F0/R0. (**c**) Immunostaining of TRA1-81 and NANOG in the corrected iPSC. Scale bar, 100 μm. (**d**) Representative Na current traces recorded on HEK293T cell transfected with WT-Nav1.1 (*n=*8) and Q1923R (*n=*5). (**e**) Western blot analysis of cell surface expression of WT-Nav1.1 and Q1923R in transfected HEK293T cells. Negative control indicates the untransfected HEK293T cells, and two independent assays were performed. (**f–g**) Co-immunostaining of MAP2 with glutamatergic neuron-specific marker glutamate (Glu) and GABAergic neuron-specific marker GABA. Scale bar, 100 μm. (**h**) Percentage of each neuronal subtype out of total MAP2-positive neurons.

We previously reported the production of an epileptic iPSC line by introducing a mutation site into normal human iPSC line through TALEN-mediated homologous recombination.^30^ In this study, the same method was used to correct the missense mutation A5768G in patient iPSCs. To correct the mutation site without introducing an exogenous sequence into the coding region, the TALEN vector was designed to cleave in the 3′ untranslated regions of *SCN1A,* and the 5′ arm homology containing the wild-type locus that was amplified from the genetic DNA of control iPSCs was cloned into the targeting donor (Figure 1a). Upon cleavage by TALENs and subsequent homologous recombination with the donor template, the epilepsy-causing A5768G mutation in patient iPSCs should be replaced by the wild-type locus in the donor template. After electroporation, the correctly targeted clones were selected by G418 and Sanger sequencing using primers F0/R0. Sequencing of the PCR products revealed the replacement of the mutation site by the wild-type locus in one of the G418-resistant clones (Figure 1b). This corrected iPSC clone maintained normal morphology and expressed the pluripotent markers TRA-1-81 and NANOG (Figure 1c). Analyses of potential off-target event were performed as we described before,^30^ and the results revealed the absence of mutations at our predicted off-target sites.

Electrophysiological study performed on HEK293T cells that were transiently transfected with Q1923R complementary DNA showed that the mutant channels exhibited little or no current compared with wild-type Nav1.1 (WT-Nav1.1; Figure 1d). Western blot analysis of the transfected cells showed similar amounts of plasma membrane expression of Q1923R and WT-Nav1.1 (Figure 1e), suggesting a normal expression of Q1923R on the transfected cell surface. Thus, the mutation was identified as loss-of-function.

### Neuronal differentiation generated a mixture of GABAergic neurons, glutamatergic neurons and glial cells

In the present study, a dorsal neuronal differentiation method was adopted to generate neuronal cultures comprising GABAergic neurons, glutamatergic neurons and glial cells. After self-renewing factors were removed, the iPSCs were cultured in chemically defined medium and differentiated predominantly into neuroepithelia,^34, 35^ which then, in the absence of exogenous morphogens, further differentiated into Pax6-expressing dorsal fate progenitors^36, 37^ in the form of rosettes (Supplementary Figure 1f). During subsequent neuronal differentiation, the progenitor cells generated TUJ1-positive neurons and, over time, glial fibrillary acidic protein positive gliocytes (Supplementary Figure 1g). The acquired neurons included both glutamatergic (Figure 1f) and GABAergic neurons (Figure 1g), accounting for 53.20±3.13% and 35.99±1.92% of the total MAP2-positive neurons, respectively (Figure 1h). These neurons exhibited typical electrophysiological properties (Supplementary Figures 1h–j) and formed electrophysiologically functional synaptic connections (Supplementary Figure 1k). These results above showed that the iPSC lines (patient, control and corrected) could undergo normal neuronal differentiation process and lead to functional neurons by this differentiation method.

### Labeling of the GABAergic subtype by tdTomato using CRISPR/Cas9-mediated knock-in

**Figure 2:**
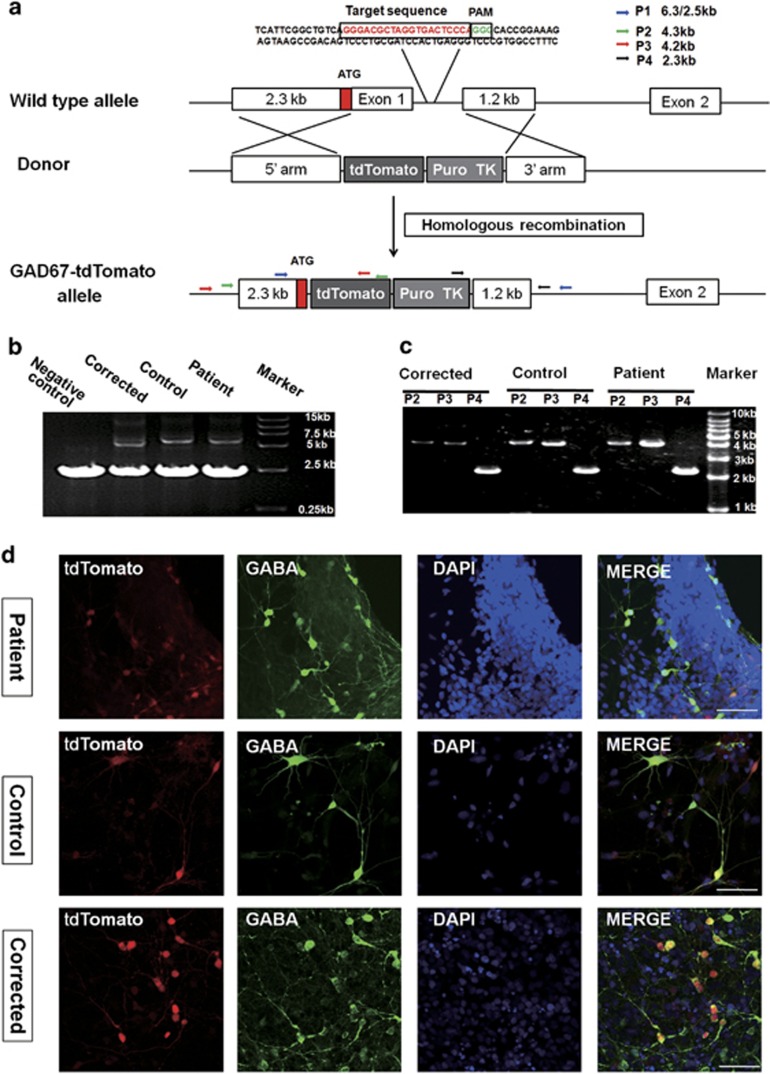
Generation of GAD67-tdTomato knock-in human iPSC lines. (**a**) Schematics of wild-type, donor and recombinant alleles of GAD67. P1–P4 indicate primer pairs 1–4. (**b**) Representative PCR analysis of puromycin-resistant clones by P1. Negative control indicates the PCR band from td^−/−^ iPSCs. (**c**) PCR analysis of puromycin-resistant clones using P2–P4. (**d**) GABA immunostaining reveals strong co-localization of tdTomato and GABA. Scale bar, 100 μm.

Because a differentiation protocol that generated both GABAergic and glutamatergic neurons was used, we need to distinguish different neuronal subtypes to investigate the electrophysiological abnormality of the Nav1.1-expressing subtype. GAD67 is a GABA-synthesizing enzyme that is specifically expressed in GABAergic neurons but not in glutamatergic neurons. Tamamaki *et al.*^38^ reported that the heterozygous knock-in of EGFP into the first exon of the GAD67 gene did not affect the function of GABAergic neurons and the acquired GAD67-GFP knock-in mice exhibited normal growth and normal lifespan. Thus, we selected GAD67-tdTomato knock-in strategy to label the GABAergic neurons. The complementary DNA of the red fluorescent protein tdTomato was targeted to the locus encoding GAD67 using CRISPR/Cas9-mediated homologous recombination (Figure 2a) in the iPSCs of patient, control and corrected. After puromycin screenings, we acquired iPSC clones with one of their chromosomes inserted with the *tdTomato* gene, and the other chromosome remaining unchanged (td^+/−^ iPSCs). This insertion was confirmed with the PCR results that two expected bands of different sizes were amplified from the genetic DNA of patient, control and corrected td^+/−^ iPSCs, respectively, using primer pair P1 (Figure 2b). Subsequent sequencing and results from other primer pairs also confirmed the correct insertion of the *tdTomato* gene and the intactness of the genome sequence flanking it (Figure 2c). To monitor the possible off-targeting introduced by Cas9-directed cleavage, 11 potential off-target sites homologous to the guide RNA-targeted site were found by BLAST search (Supplementary Table 2). These potential sites were analyzed using PCR and sequencing, and none of the 11 regions showed evidence of off-target cleavage for any of the three obtained td^+/−^ iPSCs. These obtained td^+/−^ iPSC lines expressed the pluripotent markers and were able to undergo normal neuronal differentiation and generate neurons with typical neuronal morphology and electrophysiological characteristics (data not shown). The immunostaining of neurons differentiated from the td^+/−^ iPSC lines of patient, control and corrected all showed strong co-localization patterns between tdTomato-positive neurons (td^+^ neurons) and GABA-positive neurons (Figure 2d), demonstrating that the inserted tdTomato was specifically expressed in GABAergic neurons and that this insertion did not abolish the function of GAD67 in GABA synthesis in td^+^ GABAergic neurons at the macroscopic level.

Nav1.1 was expressed in the td^+^ subtype; the sodium current density decreased and activation curve right shift in patient-derived td^+^ GABAergic neurons.

**Figure 3:**
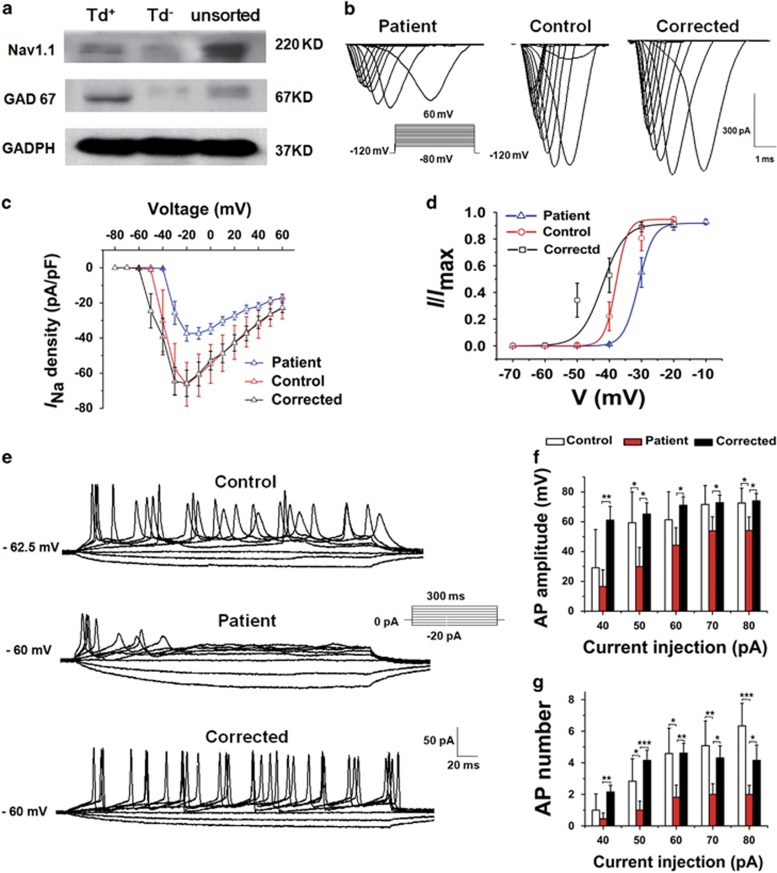
Impaired sodium channel function and action potential (AP)-firing ability in Nav1.1-expressing GABAergic neurons. (**a**) Western blot analysis of total Nav1.1 expression in td^+^ and td^−^ neurons. Unsorted indicates a mixture of td^+^ and td^−^ neurons. (**b**) Representative traces of sodium currents recorded in td^+^ neurons of patient, control and corrected. Curves of sodium current (*I*_Na_) density (**c**) and sodium activation (**d**) of patient (*n=*16), control (*n*=12) and corrected (*n*=14) td^+^ neurons (mean±s.e.m.). (**e**) Representative traces of APs recorded in td^+^ neurons of patient, control and corrected. Average AP amplitude (**f**) and firing number (**g**) for control (*n*=12), patient (*n*=11) and corrected (*n*=13) td^+^ neurons (mean±s.e.m.; *P<0.05, **P<0.01 and ***P<0.001, unpaired *t*-test).

We first conducted a flow cytometry experiment to obtain td^+^ and td^−^ neurons (5 weeks after plating, Supplementary Figure 1l). In the following western blot experiment, the td^+^ population was found expressing both GAD67 and Nav1.1, whereas the td^−^ population expressing minimal levels of both (Figure 3a). This finding suggested that Nav1.1 was expressed primarily on GABAergic neurons but rarely on glutamatergic neurons in this differentiated neuronal system.

Then, electrophysiological characteristics of total sodium channel were selectively checked on td^+^ neurons (4–5 weeks after plating, Supplementary Figure 1m and Figure 3b) and the results showed that the sodium current density (Figure 3c) for patient-derived GABAergic neurons was significantly lower than those of control and corrected. Moreover, the activation curve (Figure 3d) for patient-derived GABAergic neurons shifted rightward compared with that of control, and this shift was even larger when compared with corrected (the *V*_1/2_ for patient, control and corrected are −30.69±0.76, −37.87±1.40 and −42.14±1.78, respectively). This right shift indicated an increased threshold of the Nav channel, implying a decreased activation ability for sodium channels in patient-derived GABAergic neurons.

### AP-firing ability was impaired in patient-derived GABAergic neurons

We further tested the ability of GABAergic neurons to fire AP in response to depolarizing current pulses. AP-firing ability was mainly influenced by the number of available sodium channels, which was partially controlled by RMP in addition to the channel expression and conductance. To test how the alterations in Nav current will influence the AP-firing ability of GABAergic neurons, we recorded the APs of td^+^ neurons with similar RMPs that ranged from−60 to−70 mV (7–8 weeks after plating, Figure 3e). The average RMPs did not differ significantly among patient, control and corrected. However, the average threshold for AP initiation in patient was substantially higher than the corrected. Similar to alterations in Nav activation, the increase of threshold in patient compared with control was smaller than that compared with corrected and was not statistically significant. Clearly, the patient-derived GABAergic neurons required more depolarization current and a larger increase in membrane potential to fire APs (Table 1). In addition, the statistical data showed a reduction in the AP amplitude (Figure 3f), and AP number (Figure 3g) for each step of 300 ms current injection in patient-derived GABAergic neurons compared with both control and corrected.

### sEPSCs dominated in patient-derived neuronal network, whereas sIPSCs dominated in control and corrected neuronal networks

**Figure 4:**
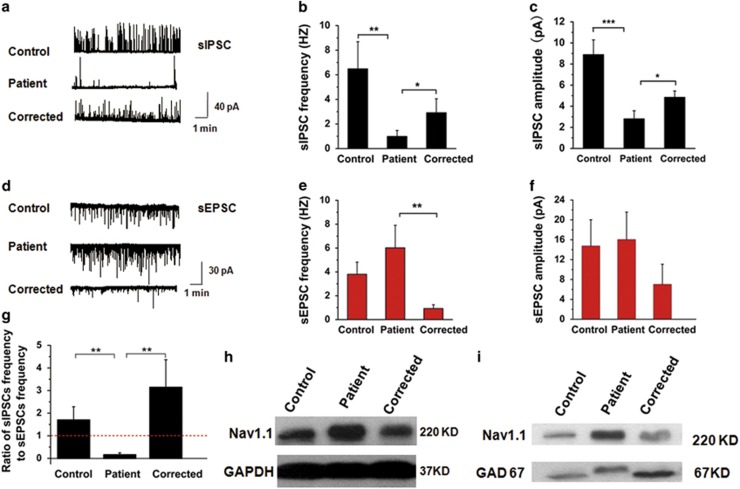
Postsynaptic activity in neuronal networks derived from td^−/−^ iPSC lines. (**a**) Representative traces of GABAergic spontaneous inhibitory postsynaptic currents (sIPSCs) recorded in neuronal networks differentiated from td^−/−^ iPSC lines. The average values (±s.e.m.) of GABAergic sIPSC frequencies (**b**) and amplitudes (**c**) recorded in neuronal networks derived from td^−/−^ iPSC lines (control, *n*=30; patient, *n*=37; corrected, *n*=30; *P<0.05, **P<0.01 and ***P<0.001, unpaired *t*-test). (**d**) Representative traces of glutamatergic spontaneous excitatory postsynaptic currents (sEPSCs) recorded in neuronal networks differentiated from td^−/−^ iPSC lines. The average values (±s.e.m.) of glutamatergic sEPSC frequencies (**e**) and amplitudes (**f**) recorded in neuronal networks derived from td^−/−^ iPSC lines (control, *n*=30; patient, *n*=37; corrected, *n*=30; *P<0.05, **P<0.01 and ***P<0.001, unpaired *t*-test). (**g**) Ratios of sIPSC frequency to sEPSC frequency show excitatory levels of neuronal networks (average values±s.e.m.; control, *n*=30; patient, *n*=37; corrected, *n*=30; *P<0.05, **P<0.01 and ***P<0.001, unpaired *t*-test). Representative immunoblots of total Nav1.1 expression relative to GAPDH (**h**) and GAD67 (**i**) in neuronal cultures differentiated from td^−/−^ iPSC lines (three replicates).

Next, we monitored spontaneous postsynaptic activity in neuronal networks (7–8 weeks after plating) differentiated from td^−/−^ iPSCs (not containing the *tdTomato* gene). In our recording condition, the recorded sIPSCs were GABAergic, which were positive and could be blocked by bicuculline; the sEPSCs were glutamatergic that were negative and could be inhibited by CNQX/MK801 (Supplementary Figures 2a and 2b). Spontaneous postsynaptic currents could not always be recorded in tested neurons; thus, we only selected and analyzed those neurons from which we could record at least one type of spontaneous postsynaptic current. The results showed that the frequency and amplitude of sIPSCs (Figures 4a–c) in patient-derived neurons were significantly lower than those of both control and corrected. However, the sEPSCs (Figures 4d–f) from patient-derived neurons did not change significantly compared with those of control and corrected, except for a higher frequency compared with corrected. When the frequency of postsynaptic currents was further analyzed, we found that the ratio of sIPSC frequency to sEPSC frequency (Figure 4g) was significantly smaller in patient-derived neurons compared with that in control and corrected, and that the control- and corrected-derived neuronal network displayed an inhibitory postsynaptic predominant state, whereas the patient-derived neurons showed a primarily excitatory state.

### Total Nav1.1 expression increased in neuronal culture generated from patient td^−/−^ iPSCs

Mutation has been reported to disrupt Nav trafficking and expression.^31, 39, 40^ In our study, the neuronal cultures differentiated from the three td^−/−^ iPSC lines (patient, control and corrected) expressed Nav1.1. However, in contrast to the decreased sodium channel function, an increase in the total Nav1.1 expression relative to GAPDH was observed in neuronal culture differentiated from patient td^−/−^ iPSCs (5 weeks after plating, Figure 4h). To eliminate the possibility that this observed difference was caused by the variations in the proportion of GABAergic neurons among neuronal cultures differentiated from different iPSC^−/−^ lines, we measured total Nav1.1 expression relative to GAD67, which was also higher in patient-derived neuronal culture compared with that in control and corrected (Figure 4i). Considering the insufficiency of Nav function in patient-derived GABAergic neurons, this upregulation might represent a compensatory response to the insufficient sodium current. Because of lacking proper method to assess the surface expression of Nav1.1 in our mixed neuronal system, further determination of whether this upregulation came from surface expression increase or *de novo* synthesized intracellular rise was unable to be made.

## Discussion

In this study, by specifically labeling GABAergic subtype in the neuronal network derived from epileptic patient iPSCs, we investigated the direct effect of *SCN1A* loss-of-function mutation on Nav characteristics in Nav1.1-expressing neuronal subtype. Our results demonstrated that this mutation influenced Nav properties in this neuronal subtype in two ways. First, the sodium current density in patient-derived GABAergic neurons decreased. Second, the Nav activation curve rightward-shifted in patient-derived GABAergic neurons. This right shift is unexpected because no such change in the activation curve was observed on transfected cells. Different Nav subtypes have different activation curves, and there are at least two other Nav subtypes expressed on GABAergic neurons (Nav1.3, Nav1.6),^16, 17^ the activation curve recorded on GABAergic neurons thus is a total effect of these different Nav subtypes. Because the activation curve of Nav1.1 is on the left relative to all other Nav subtypes expressed in the brains (Nav1.3, Nav1.6 and Nav1.2),^41^ it can be speculated that when the component of Nav current contributed by Nav1.1 reduced, this channel contributes less to the total effect of activation curve in GABAergic neurons. Therefore, the channel activation curve shifted away from the position of Nav1.1 activation curve accordingly. Measurement of AP properties in patient-derived GABAergic neurons showed that the threshold of AP enhanced, whereas the amplitude and number decreased. These alterations were in accordance with the changes in Nav characteristic, suggesting that the loss-of-function mutation in Nav1.1 has further impaired AP-firing ability, and that both aspects of the alterations of Nav are involved in the impairment of patient-derived GABAergic neuronal excitability.

The Nav activation and AP threshold in patient-derived GABAergic neurons altered significantly compared with that of corrected; however, this alteration became smaller when compared with that of control. As discussed above, genetic backgrounds can modulate disease severity.^25^ The patient and the corrected iPSC lines have the same genetic background, except for the mutation site, whereas the control iPSC line has a different genetic background. We thus speculate that this observed phenomenon may result from the modulatory effect of genetic background, and may also offer a possible explanation for the phenomenon that different affected members from this patient's family^29^ as well as some genetic epilepsy with febrile seizures plus families^24^ show phenotypic variability.

Further monitoring of the spontaneous postsynaptic currents showed that the altered amplitude and firing number of AP worked together to influence the GABAergic sIPSCs in patient-derived neuronal network because both the frequency and amplitude of sIPSCs decreased. Although the sEPSCs did not change significantly as the sIPSCs, when the frequency of both types of spontaneous postsynaptic currents were further analyzed, we found that the pattern of postsynaptic current transferred from a sIPSC-dominated state to a sEPSC-dominated state in the patient-derived neuronal network, suggesting that changes in the sIPSCs alone was sufficient to significantly affect the whole condition of spontaneous postsynaptic currents. These changes in the spontaneous postsynaptic currents may not necessarily represent an epileptic hyperexcitability; however, they reveal the physiological basis for occurrence of epilepsies and provide clinical implications for drug administration.

Antiepileptic drugs can be assigned to four categories according to their mechanisms of action: sodium channel blocker, GABA analog, synaptic vesicle protein 2A binding and multiple mechanisms.^42^ The Nav functional insufficiency of GABAergic neurons caused by the mutation was identified as the origin of disease and which finally caused insufficiency of GABAergic activity as well as the transition from inhibition- to excitation-dominated state in the patient-derived neuronal network. Therefore, to prevent from further attenuating function of the Nav channel on GABAergic neurons, treatment with sodium channel-blocking antiepileptic drugs should be avoided in this patient's family. Although sodium channel blocker can also act on Nav channel on glutamatergic neurons and then have an antiepileptic role during seizure occurence, sEPSCs recorded in our system did not significantly change in the patient, suggesting that drug acting on glutamatergic neurons is not a must or urgency. Our results showed that the significantly changed neuronal subtype is GABAergic neurons, which suggested that the selection of GABA analog is a more niche-targeting strategy to treat epilepsy in this patient's family as well as in other patients with *SCN1A* loss-of-function.

Different strategies have been reported to generate epileptic mouse models to mimic the loss-of-function mutation,^18, 21, 22^ which led to Nav1.1 deletion in GABAergic neurons of different brain regions, subtypes, or to different extents. Although with different severities, these models showed epileptic phenotype, suggesting that Nav1.1-expressing GABAergic neurons of different subtypes and regions may have a similar role in epileptogenesis caused by Nav1.1 loss-of-function. Therefore, study conducted on neocortex GABAergic neurons of PEFS+ patient in the present project might represent the situation of other Nav1.1-positive GABAergic subtypes, and could provide a possible explanation for the whole type of epilepsy caused by loss-of-function mutation.

Both sIPSCs and sEPSCs were measured, and their relative value was used as an index to assess the excitability of neuronal network in this work. Using this index, we believe we showed for the first time that the neuronal network derived from healthy iPSCs was predominated by inhibitory postsynaptic activity. This finding was interesting because glutamatergic neurons composed the majority of this neuronal network. Both loss-of-function and gain-of-function mutations in Nav1.1 channels can cause epilepsy. It appears that gain-of-function mutations enhance the function of GABAergic neurons and prevent epilepsy occurrence. However, we found that sIPSC was stronger than sEPSC in normal neuronal networks, which implied that GABAergic neurons made more synapses than excitatory neurons in this system, and thus it was more likely to form synapses between two GABAergic neurons than between GABAergic and glutamatergic neurons. Accordingly, when the Nav function in GABAergic neurons was enhanced, the first or more extensively influenced downstream neurons were more likely to be GABAergic neurons rather than glutamatergic neurons, and then the GABAergic neurons would be inhibited to an extent greater than the glutamatergic neurons. In this condition, gain-of-function mutations most likely cause the same result as loss-of-function mutations.

Jiao *et al.*^33^ previously studied the glutamatergic neurons differentiated from patient iPSCs with the same mutation, Q1923R, and found no significant changes in their electrophysiological properties. This result is complement with our finding that GABAergic neuron is the subtype that is directly influenced by this mutation and the origin of disease. In addition, Liu et *al.*^19^ reported another work conducted using iPSCs derived from a Dravet syndrome patient who bears a different mutation site that was also identified exogenously as loss-of-function. However, in their work, they showed increased Nav current density and spontaneous bursting in GABAergic neurons, which are conflicted with our findings. The reason for this conflict might be that the GABAergic neurons recorded in the two studies originate from different lineages. There are two distinct lineages of GABAergic neurons that exist in the human neocortex, one originates from progenitors of the dorsal forebrain and the other from the ventral forebrain.^43^ Nav1.1 is not expressed in all GABAergic subtypes, and GABAergic neurons from different lineages may have different Nav1.1 expression. Thus, with no information regarding the Nav1.1 expression pattern in their recorded neurons, it was difficult to determine whether the observed phenotype was a direct result of *SCN1A* mutation and a reason for the seizure, or was the intermediate stage of seizure, or even was a post-seizure effect.

Parent *et al.*^15^ have pointed that, for complex network disorders, such as epilepsies and psychiatric disturbances, iPSCs have the same limitations inherent to all reductionist *in vitro* approaches regarding the failure to recapitulate complex, three-dimensional neural circuitry. In this project, we obtained neuronal system containing both types of neurons that might be involved in epilepsy and recorded the synaptic activities in this simple neuronal network. However, our data simply show connections of a neuron to its upper stream neuron, and the preparation in the present study is still far from any real neuronal network that one can observe in rodents. Thus, the observation acquired in this study can only present the phenomenon of an *in vitro* system.

In conclusion, the mutation Q1923R caused not only a reduction in amount of Nav currents but also a rightward shift in the Nav activation curve. This finding fills the gap of our knowledge regarding the relationship between mutation effect recorded on exogenously transfected cells and Nav1.1-expressing neurons that, besides directly influencing the amount of Nav currents, the exogenously identified mutant that produces no current can also influence Nav channel properties such as activation. Mechanistically, the two alterations in Nav further influenced the AP-firing ability in patient-derived GABAergic neurons and led to a weakened sIPSC as well as the shift of spontaneous postsynaptic input from the inhibition- to excitation-dominated state in patient-derived neuronal network. Our study reveals the physiological basis underlying epileptogenesis that is caused by *SCN1A* loss-of-function mutation and provides practical instruction for clinical drug administration.

## Figures and Tables

**Table 1:** Threshold of action potentials for td^+^ GABAergic neurons with resting membrane potentials ranging from −70 to −60 mV

*(mV)*	*Patient (*n*=12)*	*Control (*n*=12)*	P_*1*_	*Corrected (*n*=13)*	P_*2*_
RMP	−63.69±0.86	−64.33±0.99	0.48	−63.00±1.02	0.80
Threshold	−30.07±1.68	−34.48±2.26	0.073	−39.06±1.73	0.00076
MP increase	33.28±1.87	29.85±2.25	0.13	23.93±1.56	0.00044

Abbreviations: MP, membrane potential; *P*_1_, the difference between patient and control; *P*_2_, the difference between patient and corrected; RMP, resting membrane potential.

Average values±s.e.m., unpaired *t*-test.

## References

[bib1] Catterall WA, Kalume F, Oakley JC. NaV1.1 channels and epilepsy. J Physiol 2010; 588: 1849–1859. 20194124 10.1113/jphysiol.2010.187484 PMC2901973

[bib2] Escayg A, MacDonald BT, Meisler MH, Baulac S, Huberfeld G, An-Gourfinkel I et al. Mutations of SCN1A, encoding a neuronal sodium channel, in two families with GEFS+. Nat Genet 2000; 24: 343–345. 10742094 10.1038/74159

[bib3] Dravet C. Dravet syndrome history. Dev Med Child Neurol 2011; 53: 1–6. 10.1111/j.1469-8749.2011.03964.x 21504424

[bib4] Steinlein OK. Genetic mechanisms that underlie epilepsy. Nat Rev Neurosci 2004; 5: 400–408. 15100722 10.1038/nrn1388

[bib5] Watanabe M, Fujiwara T, Yagi K, Seino M, Higashi T. Intractable childhood epilepsy with generalized tonic–clonic seizures. J Jpn Epil Soc 1989; 7: 96–105.

[bib6] Margolis JM, Chu BC, Wang ZJ, Copher R, Cavazos JE. Effectiveness of antiepileptic drug combination therapy for partial-onset seizures based on mechanisms of action. JAMA Neurol 2014; 71: 985–993. 24911669 10.1001/jamaneurol.2014.808

[bib7] Lossin C, Wang DW, Rhodes TH, Vanoye CG, George AL Jr. Molecular basis of an inherited epilepsy. Neuron 2002; 34: 877–884. 12086636 10.1016/s0896-6273(02)00714-6

[bib8] Lossin C, Rhodes TH, Desai RR, Vanoye CG, Wang D, Carniciu S et al. Epilepsy-associated dysfunction in the voltage-gated neuronal sodium channel SCN1A. J Neurosci 2003; 23: 11289–11295. 14672992 10.1523/JNEUROSCI.23-36-11289.2003 PMC6740520

[bib9] Sugawara T, Tsurubuchi Y, Fujiwara T, Mazaki-Miyazaki E, Ngata K, Montal M et al. Nav1.1 channels with mutations of severe myoclonic epilepsyin infancy display attenuated currents. Epilepsy Res 2003; 54: 201–207. 12837571 10.1016/s0920-1211(03)00084-6

[bib10] Rhodes TH, Lossin C, Vanoye CG, Wang DW, George AL Jr. Noninactivating voltage-gated sodium channels in severe myoclonic epilepsy of infancy. Proc Natl Acad Sci USA 2004; 101: 11147–11152. 15263074 10.1073/pnas.0402482101 PMC503754

[bib11] Ohmori I, Kahlig KM, Rhodes TH, Wang DW, George AL Jr. Nonfunctional SCN1A is common in severe myoclonic epilepsy of infancy. Epilepsia 2006; 47: 1636–1642. 17054685 10.1111/j.1528-1167.2006.00643.x

[bib12] Mantegazza M, Rusconi R, Scalmani P, Avanzini G, Franceschetti S. Epileptogenic ion channel mutations: from bedside to bench and, hopefully, back again. Epilepsy Res 2010; 92: 1–29. 20828990 10.1016/j.eplepsyres.2010.08.003

[bib13] Takahashi K, Tanabe K, Ohnuki M, Narita M, Ichisaka T, Tomoda K et al. Induction of pluripotent stem cells from adult human fibroblasts by defined factors. Cell 2007; 131: 861–872. 18035408 10.1016/j.cell.2007.11.019

[bib14] Shi Y, Kirwan P, Livesey FJ. Directed differentiation of human pluripotent stem cells to cerebral cortex neurons and neural networks. Nat Protoc 2012; 7: 1836–1846. 22976355 10.1038/nprot.2012.116

[bib15] Parent JM, Anderson SA. Reprogramming patient-derived cells to study the epilepsies. Nat Neurosci 2015; 18: 360–366. 25710838 10.1038/nn.3944 PMC4483308

[bib16] Kalume F, Yu FH, Westenbroek RE, Scheuer T, Catterall WA. Reduced sodium current in Purkinje neurons from Nav1.1 mutant mice: implications for ataxia in severe myoclonic epilepsy in infancy. J Neurosci 2007; 27: 11065–11074. 17928448 10.1523/JNEUROSCI.2162-07.2007 PMC6672849

[bib17] Yu FH, Mantegazza M, Westenbroek RE, Robbins CA, Kalume F, Burton KA et al. Reduced sodium current in GABAergic interneurons in a mouse model of severe myoclonic epilepsy in infancy. Nat Neurosci 2006; 9: 1142–1149. 16921370 10.1038/nn1754

[bib18] Ogiwara I, Iwasato T, Miyamoto H, Iwata R, Yamagata T, Mazaki E et al. Nav1.1 haploinsufficiency in excitatory neurons ameliorates seizure-associated sudden death in a mouse model of Dravet syndrome. Hum Mol Genet 2013; 22: 4784–4804. 23922229 10.1093/hmg/ddt331 PMC3820136

[bib19] Liu Y, Lopez-Santiago LF, Yuan Y, Jones JM, Zhang H, O'Malley HA et al. Dravet syndrome patient-derived neurons suggest a novel epilepsy mechanism. Ann Neurol 2013; 74: 128–139. 23821540 10.1002/ana.23897 PMC3775921

[bib20] Ogiwara I, Miyamoto H, Morita N, Atapour N, Mazaki E, Inoue I et al. Nav1.1 localizes to axons of parvalbumin-positive inhibitory interneurons: a circuit basis for epileptic seizures in mice carrying an Scn1a gene mutation. J Neurosci 2007; 27: 5903–5914. 17537961 10.1523/JNEUROSCI.5270-06.2007 PMC6672241

[bib21] Cheah CS, Yu FH, Westenbroek RE, Kalume FK, Oakley JC, Potter GB et al. Specific deletion of NaV1.1 sodium channels in inhibitory interneurons causes seizures and premature death in a mouse model of Dravet syndrome. Proc Natl Acad Sci USA 2012; 109: 14646–14651. 22908258 10.1073/pnas.1211591109 PMC3437823

[bib22] Dutton SB, Makinson CD, Papale LA, Shankar A, Balakrishnan B, Nakazawa K et al. Preferential inactivation of Scn1a in parvalbumin interneurons increases seizure susceptibility. Neurobiol Dis 2013; 49: 211–220. 22926190 10.1016/j.nbd.2012.08.012 PMC3740063

[bib23] Westenbroek RE, Merrick DK, Catterall WA. Differential subcellular localization of the RIand RIINa+ channel subtypes in central neurons. Neuron 1989; 3: 695–704. 2561976 10.1016/0896-6273(89)90238-9

[bib24] Scheffer IE, Berkovic SF. Generalized epilepsy with febrile seizures plus. A genetic disorder with heterogeneous clinical phenotypes. Brain 1997; 120: 479–490. 9126059 10.1093/brain/120.3.479

[bib25] Singh R, Andermann E, Whitehouse WP, Harvey AS, Keene DL, Seni MH et al. Severe myoclonic epilepsy of infancy: extended spectrum of GEFS+. Epilepsia 2001; 42: 837–844. 11488881 10.1046/j.1528-1157.2001.042007837.x

[bib26] Miller JC, Tan S, Qiao G, Barlow KA, Wang J, Xia DF et al. A TALE nuclease architecture for efficient genome editing. Nat Biotechnol 2011; 29: 143–148. 21179091 10.1038/nbt.1755

[bib27] Mali P, Yang L, Esvelt KM, Aach J, Guell M, DiCarlo JE et al. RNA-guided human genome engineering via Cas9. Science 2013; 339: 823–826. 23287722 10.1126/science.1232033 PMC3712628

[bib28] Gaj T, Gersbach CA, Barbas CF 3rd. ZFN, TALEN, and CRISPR/Cas-based methods for genome engineering. Trends Biotechnol 2013; 31: 397–405. 23664777 10.1016/j.tibtech.2013.04.004 PMC3694601

[bib29] Shi YW, Yu MJ, Long YS, Qin B, He N, Meng H et al. Mosaic SCN1A mutations in familial partial epilepsy with antecedent febrile seizures. Genes Brain Behav 2012; 11: 170–176. 22151702 10.1111/j.1601-183X.2011.00756.x

[bib30] Chen WJ, Liu JX, Zhang LM, Xu HJ, Guo XG, Deng SH et al. Generation of the SCN1A epilepsy mutation in hiPS cells using the TALEN technique. Sci Rep 2014; 4: 5404. 24953032 10.1038/srep05404 PMC4066246

[bib31] Thompson CH, Porter JC, Kahlig KM, Daniels MA, George AL Jr. Nontruncating SCN1A mutations associated with severe myoclonic epilepsy of infancy impair cell surface expression. J Biol Chem 2012; 287: 42001–42008. 23086956 10.1074/jbc.M112.421883 PMC3516746

[bib32] Verret L, Mann EO, Hang GB, Barth AM, Cobos I, Ho K et al. Inhibitory interneuron deficit links altered network activity and cognitive dysfunction in Alzheimer model. Cell 2012; 149: 708–721. 22541439 10.1016/j.cell.2012.02.046 PMC3375906

[bib33] Jiao J, Yang Y, Shi Y, Chen J, Gao R, Fan Y et al. Modeling Dravet syndrome using induced pluripotent stem cells (iPSCs) and directly converted neurons. Hum Mol Genet 2013; 22: 4241–4252. 23773995 10.1093/hmg/ddt275

[bib34] Zhang SC, Wernig M, Duncan ID, Brustle O, Thomson JA.*In vitro* differentiation of transplantable neural precursors from human embryonic stem cells. Nat Biotechnol 2001; 19: 1129–1133. 11731781 10.1038/nbt1201-1129

[bib35] Pankratz MT, Li XJ, Lavaute TM, Lyons EA, Chen X, Zhang SC. Directed neural differentiation of human embryonic stem cells via an obligated primitive anterior stage. Stem Cells 2007; 25: 1511–1520. 17332508 10.1634/stemcells.2006-0707 PMC2743478

[bib36] Johnson MA, Weick JP, Pearce RA, Zhang SC. Functional neural development from human embryonic stem cells: accelerated synaptic activity via astrocyte coculture. J Neurosci 2007; 27: 3069–3077. 17376968 10.1523/JNEUROSCI.4562-06.2007 PMC2735200

[bib37] Li XJ, Zhang X, Johnson MA, Wang ZB, Lavaute T, Zhang SC. Coordination of sonic hedgehog and Wnt signaling determines ventral and dorsal telencephalic neuron types from human embryonic stem cells. Development 2009; 136: 4055–4063. 19906872 10.1242/dev.036624 PMC2778748

[bib38] Tamamaki N, Yanagawa Y, Tomioka R, Miyazaki J, Obata K, Kaneko T. Green fluorescent protein expression and colocalization with calretinin, parvalbumin, and somatostatin in the GAD67-GFP knock-in mouse. J Comp Neurol 2003; 60–79. 10.1002/cne.10905 14574680

[bib39] Abriel H, Kass RS. Regulation of the voltage-gated cardiac sodium channel Nav1.5 by interacting proteins. Trends Cardiovasc Med 2005; 15: 35–40. 15795161 10.1016/j.tcm.2005.01.001

[bib40] Rusconi R, Scalmani P, Cassulini RR, Giunti G, Gambardella A, Franceschetti S et al. Modulatory proteins can rescue a trafficking defective epileptogenic Nav1.1 Na+ channel mutant. J Neurosci 2007; 27: 11037–11046. 17928445 10.1523/JNEUROSCI.3515-07.2007 PMC6672853

[bib41] Gilchrist J, Dutton S, Diaz-Bustaman M, McPherson A, Olivares N, Kalia J et al Nav1.1 modulation by a novel triazole compound attenuates epileptic seizures in rodents. ACS Chem Biol 2014; 9: 1204–1212. 24635129 10.1021/cb500108p PMC4027953

[bib42] St Louis EK. Truly ‘rational' polytherapy: maximizing efficacy and minimizing drug interactions, drug load, and adverse effects. Curr Neuropharmacol 2009; 7: 96–105. 19949567 10.2174/157015909788848929 PMC2730011

[bib43] Letinic K, Zoncu R, Rakic P. Origin of GABAergic neurons in the human neocortex. Nature 2002; 417: 645–649. 12050665 10.1038/nature00779

