# Supplementary material for: CRISPR/Cas9 facilitates investigation of neural circuit disease using human iPSCs: mechanism of epilepsy caused by an SCN1A loss-of-function mutation
Source: Transl Psychiatry. 2016 Jan 5;6(1):e703–. doi: 10.1038/tp.2015.203 (PMC5068877; doi:10.1038/tp.2015.203)
Supplement: Supplementary Information [file tp2015203x1.doc]

**Supplementary Materials and Methods**

*Ethics statement.* The epileptic patient skin sample was obtained from the Second Affiliated Hospital of Guangzhou Medical University, and written informed consent was obtained from the patient. The research procedure was approved by the institutional review board of the Second Affiliated Hospital of Guangzhou Medical University, and by the human subject research ethics committee of Guangzhou Institutes of Biomedicine and Health.

*hiPSC neuronal differentiation.* In the present study, a dorsal neuronal differentiation method was adopted. This method generates neuronal culture with a larger proportion of glutamatergic subtype than GABAergic subtype, which is similar to the composition in human neocortex where there are more glutamatergic neurons than GABAergic neurons. And meanwhile, the proportion of GABAergic neurons is large enough for electrophysiological study and Nav1.1 expression analysis. The differentiation procedure followed a previously described method (34-37) with minor adjustments. iPSCs were cultured in 6-well plates for 7 days to reach 100% confluence, then split up using a 1-ml pipette after 5 min incubation with dispase (1 mg/ml; Invitrogen) and rinsed 3 times with 1 ml DMEM/F12. The pieces of iPSCs were collected and suspension-cultured in DMEM/F12 supplemented with 20% KnockOut Serum Replacement, 1% L-glutamine, 1% non-essential amino acids, 0.1% beta-mercaptoethanol, 5 µM TGF-bRIkinase inhibitor VI (Invitrogen), and 5 µM dorsomorphin (Sigma) for 4 days to form embryonic bodies (EBs). On day 5, the medium was changed to DMEM/F12 supplemented with 1% N2 (Invitrogen), 1% NEAA (Invitrogen), and 2 μg/ml heparin sodium. On day 7, the EBs were transferred onto Matrigel-coated 6-well plates for adherent culture. On day 15, neural tube-like rosettes were selected under a microscope and transferred into NSC maintenance medium composed of 2% B27 (Invitrogen), 1% N2, 20 ng/ml bFGF ([PeproTech](http://www.baidu.com/link?url=ng2a7mXS6ZvnRIUY927G4GZ9zd6_qIWeq8BY1sBIWFc6S5GO29FGyrry0fje2H-9)), and 20 ng/ml EGF ([PeproTech](http://www.baidu.com/link?url=ng2a7mXS6ZvnRIUY927G4GZ9zd6_qIWeq8BY1sBIWFc6S5GO29FGyrry0fje2H-9)) in DMEM/F12. Neurospheres of 200–300 μm were passaged by dissociating and blowing into single cells with accutase (Sigma). For neuronal differentiation, the single cells were seeded onto Matrigel-coated coverslips in wells or cell culture plates in neural differentiation medium containing 2% B27, 1% N2, 1 µM cAMP, 10 ng/ml BDNF, and 5 ng/ml GDNF in DMEM/F12. The neuronal culture was fed every 2 days.

*Transfection.* Transient expression of human Nav1.1 was performed on HEK293 cells. The cells were co-transfected with plasmids encoding wild-type or mutant NaV1.1, human sodium channel β1, and β2 subunits (10:1:1) using Effectence Transfection Reagent (QIAGEN) following the manufacturer’s instructions. All experiments examining the expression and electrophysiological function of the mutant were performed 24h after transfection.

***Flow cytometry.*** The neuronal cells differentiated from iPSCs that carried the inserted gene of tdTomato were incubated with accutase (Sigma) for 12 min at room temperature to dissociate neurons from neuronal networks. Single cells were harvested and washed twice in PBS. After filtered with Falcon 40µm cell strainer (BD Falcon), the cell suspensions (5×10^6^) were sorted by a MoFlo Astrios flow cytometer (MoFlo Astrios). Control samples were collected from neuronal cells differentiated from iPSCs without the inserted gene of tdTomato.

*Protein extraction and Western blot.* The cell surface protein of transfected HEK293 cells was extracted using the Membrane Protein Extraction Reagent Kit (Keygen) according to the manufacturer’s instructions. The total protein of differentiated neuronal cells was obtained using Lysis Buffer (Beyotime). Protein concentration was determined with BCA Protein Assay Kit (Pierce). Western blot analysis was performed as described previously (Thompson et al., 2012). The primary antibodies included rabbit anti-Nav1.1 (1:200; Abcam; ab24820), rabbit anti-GAD67 (1:2000; Abcam; ab108626), and rabbit anti-GAPDH (1:5000; Bioworld; AP0063). The primary antibodies were detected with horseradish peroxidase-linked secondary antibodies (goat anti-rabbit IgG, ProteinTech Group) for 2 h at room temperature. Final detection was accomplished using western blot luminal reagents47 (ProteinTech Group).

*Karyotype and teratomas analysis.* Karyotype analysis was performed using G-banding chromosome analysis. For teratomas, cells from a confluent 10-cm plate were harvested by 1 mg/ml dispase digestion, resuspended in Matrigel, and injected subcutaneously into NOD/SCID mice. Eight weeks after injection, the teratomas were dissected, paraffin embedded and stained with hematoxylin/eosin. 2 mice were injected for patient iPSC teratoma formation assay. All animal experiments in this study were following Guangzhou Institutes of Biomedicine and Health Protocol for IACUC.

*qRT-PCR.* Total RNA was isolated from fibroblasts, iPSC clones, and skin fibroblasts after the retroviral infections using TRIzol (Invitrogen) and reversely transcribed according to the manufacturer’s protocol (TaKaRa). Quantitative real-time PCR was performed using a Thermal Cycler Dice Real Time System (ABI7300, ABI) and SYBR Green Premix EX Taq^TM^ (TaKaRa). Beta-actin was used for normalizing gene expression levels.

*Immunofluorescence.* iPSCs or differentiated neuronal cells were plated onto matrigel-coated glass coverslips. The cells were fixed for 15 min at room temperature with 4% paraformaldehyde in PBS followed by three times wash with PBS. The cells were then incubated with primary antibody diluted in PBS containing 0.5% Triton X-100 and 10% normal goat serum. Afterwards， the cells were rinsed three times with 0.05% PBST and incubated for 1~2 h at room temperature with secondary antibody diluted in PBS containing 5% normal goat serum in dark. Wells were washed three times with 0.05% PBST and incubated with DAPI (0.1mg/ml in 1X PBS) at room temperature for 10 min. Finally, the coverslips containing cells were covered with anti-fade mounting medium (Beyotime) and observed by laserscanning confocal microscopy (Zeiss 710 NLO, Germany). Primary antibodies used were as follows: rabbit anti-nestin (1:200; abcam; ab105389), mouse anti-PAX-6 (1:500; Millpore; MAB5554), rabbit anti-TUJ1(1:200; Millipore; 04-1049), mouse anti-MAP2 (1:400; Millipore; MAB3418), mouse anti-GPAF(1:100,0; Millipore; MAB360), rabbit anti-GABA (1:100,0; Sigma; A2052), rabbit anti-Glutamate (1:500,0; Sigma; G6642), Rabbit anti-Nanog (1:100,0; Cell Signaling; 4903s), mouse anti-TRA-1-81 (1:100,0; Cell Signaling; 4745s). Secondary antibodies used: goat-anti mouse Alexa 555 (1:200; Cell Signaling Technology; 4409) and goat anti-rabbit Alexa 488 (1:100,0; Cell Signaling Technology; 4412).

**Figure Legends**

**Supplementary Figure 1.** Patient iPSCs exhibit hallmarks of pluripotency and human iPSC neural differentiation. (**a**) Immunostaining shows pluripotency markers of TRA1-81 and NANOG in patient iPSCs. Scale bar, 100 µm. qRT-PCR analysis show the activation of endogenous pluripotency associated genes (**b**) and the inactivation of four transgenes (**c**) used for reprogramming in patient iPSCs. PSF, patient primary skin fibroblasts. SIF, skin fibroblasts after the retroviral infection. Gene expression levels are normalized to β-actin and are presented as mean ± S.E.M., *n*=3. Gene expression levels are quantified by the ΔΔCt method. (**d**) Karyotype analysis of patient iPSC shows normal karyotype. (**e**) hematoxylin/eosin staining of teratomas derived from patient iPSCs. Scale bar, 100 µm. (**f**) Immunostaining of neural progenitors in the form of rosette shows the expression of PAX 6 and nestin. Scale bar, 100 µm. (**g**) Neuronal culture derived from human iPSCs shows neuron-specific marker TUJ1 and [astrocyte](http://www.iciba.com/astrocyte)-specific marker GFAP. Scale bar, 100 µm. Current-clamp recording of induced action potentials (**h**) and spontaneous action potentials (**i**). (**j**) Representative recordings of voltage-gated ion channels show inward sodium/calcium currents and outward potassium current. (**k**) Representative sIPSCs and sEPSCs traces detected when clamped at 0 mV and -65 mV respectively. (**l**) Flow cytometry profile showing continuous expression of tdTomato, and division of neurons into the td^+^ and td^-^ subpopulations. The acquired td^+^ population exhibits a purity of 95.81% (right panel) (**m**) Bright field image shows td^+^ GABAergic neuron recording under fluorescence microscopy.

**Supplementary Figure 2.** (**a**) Trace records of GABAergic sIPSCs in neuronal networks derived from td^-/-^iPSC lines (top panel). The current can be partially blocked by 1 µM TTX (middle panel) and completely blocked by 10 µM bicuculline (Bic) (bottom panel). (**b**) Trace recordings of glutamatergic sEPSCs in neuron network differentiated from td^-/-^iPSC lines (top panel), which can be partially blocked by 1 µM TTX (middle panel) and completely abolished by 10 µM CNQX/MK801 (bottom panel).

**Tables**

**Supplementary Table 1．**Sequences and primers related to [CRISPR](http://www.baidu.com/link?url=mxzRj4m1y4KgIVLPX7sXGdC2irgTp8Pn0m3Ggwgxxn1rSCkFkF9RUQXhJ-ZnSzsK18JKNDWY2JFfBhVLhuA-1Y11OectYy0zVCWa4dOXIRC)/Cas9- and TALNE-mediated genome editing

| **Name** | **Sequences** |
| --- | --- |
| [**CRISPR**](http://www.baidu.com/link?url=mxzRj4m1y4KgIVLPX7sXGdC2irgTp8Pn0m3Ggwgxxn1rSCkFkF9RUQXhJ-ZnSzsK18JKNDWY2JFfBhVLhuA-1Y11OectYy0zVCWa4dOXIRC)**/Cas9** |  |
| GAD67 gRNA-forward (5'→3') | *CACC*  GGGACGCTAGGTGACTCCCA |
| GAD67 gRNA-reverse (5'→3') | *AAAC*  TGGGAGTCACCTAGCGTCCC |
| 5’ arm primer-forward (5'→3') | *TTCGGGATCCGGCGCGCC* AAACTGTAATTCCTCCATGGTC |
| 5’ arm primer-reverse (5'→3') | *TTTTGGATCCC* ATCAGCTCGGTCGTCCCGGGGCCTCTAC |
| 3’ arm-primer-forward (5'→3') | *TTTTGCGGCCGC* CATCAAGCTATTGACGAGTTAG |
| 3’ arm- primer-reverse (5'→3') | *TTTTGCGGCCGC*  TATTGTCTGCAAGGGAGG |
| p1- forward (5'→3') | TCGCTGCCTTTCCTCCCTCTTGTCTC |
| p1- reverse (5'→3') | CCTGGCGGATAAAGAGCTATTTGGACCTG |
| P2- forward (5'→3') | CTTCTCCCAACGCAGCGGTTCTTT |
| P2- reverse (5'→3') | GCGCCGTTTGGACAAACCACAACT |
| P3- forward (5'→3') | TTTCTCTTTCAACGCATTTTTACG |
| P3- reverse (5'→3') | AAACCTCTACAAATGTGGTATGGC |
| P4- forward (5'→3') | GCATCGCATTGTCTGAGTAGGTGT |
| P4- reverse (5'→3') | CCTTCCAATCTCTCAAAGCAAACC |
| **TALEN** |  |
| TALEN binding site-forward (5'→3', in gray) | CACTACCAGCTGACACTGCTGAAGATAAGATGCACAATGGCTAGTCAG |
| TALEN binding site-reverse (3'→5', in gray) | GTGATGGTCGACTGTGACGACTTCTATTCTACGTGTTACCGATCAGTC |
| 5’ arm primer-forward (5'→3') | *TTTTGGTACCCG* ACGCTGCTCTTTGCTTTG |
| 5’ arm primer-reverse (5'→3') | *TTTGAATTCGC* GTCAGCTGGTAGTGAGAACAG |
| 3’ arm primer-forward (5'→3') | *TTTTGGATCC GC*  TAAGATGCACAATGGCTAG |
| 3’ arm primer-reverse (5'→3') | *TTTTGCGGCCGCGT* ATAGGTTAAGCAGTGTGTT |
| F0 -forward (5'→3') | TACCCTGTTCCGAGTGATCCGTCTT |
| R0 –reverse (5'→3') | CCCGGTCATAGGAAGGTGGACAA |

Sequences in italics refer to restriction enzyme cutting sites or [protective bases](http://www.sciencedirect.com/science/article/pii/0022391373900528).

**Supplementary Table 2.** Sequence information of gRNA and potential off- target sites.

| Featured sites | locus | Sequence (5'-3') |  |
| --- | --- | --- | --- |
|  |  | 20bp | PAM(3bp) |
| Target site | chr2:+171675350 | GGGA CGCT AGGT GACT CCCA | GGG |
| OT1 (HBD) | chr7:-155671969 | AGAA CACT AGGT GACT CCCA | AAG |
| OT2(Arfaptin-2) | chr1:-208338473 | GGGA GGCC AGGT GACT GCCA | TGG |
| OT3 (unknown) | chr16:-3162431 | TGGC CGGG AGGT GACT CCCA | GAG |
| OT4 (unknown) | chr6:+40324247 | GGGT GGGG AGGT GACT CCCA | GGG |
| OT5 | chr7:-4768997 | GGGA CCCG TGGT GACT CCCA | CAG |
| OT6 | chr16:-50420819 | AAGC CGCT TGGT GACT CCCA | GGG |
| OT7 | chr10:+134058143 | TGGA TGCC TGGT GACT CCCA | GGG |
| OT8 | chr7:-34077118 | GGTC AGCT AGAT GACT CCCA | AAG |
| OT9 | chr3:+47465630 | GGGC TGCT GAGT GACT CCCA | CAG |
| OT10 | chr8:-97057196 | GGAA GACT ACGT GACT CCCA | GAG |
| OT11 | chr10:+50380916 | GGCA CTCC ACGT GACT CCCA | CAG |

OT, Off-target site. The eleven potential off-target sites of gRNA were identified by online tools (http://crispr.mit.edu/). PAM, the trinucleotide (5'-NGG-3') protospacer adjacent motif. The mismatch nucleotides to the on-target sequences are indicated by gray.

**Supplementary Table 3.** Primers used to test potential off- target sites of gRNA.

| **Name** | **Sequences (**5'-3'**)** |
| --- | --- |
| OT1 | *forward*：TCACCTTATCCCTGGACACCT  *reverse*： CAAAGATGTCAGCCCATTCCT |
| OT2 | *forward*：GGAAGTACTTAGCAGGCGCT  *reverse*： GGCGAACAAAATCGTGGAGAG |
| OT3 | *forward*：AGAGGGCCCTTGACTAGGAG  *reverse*： AAGCCTTCCACCTTTGACCC |
| OT4 | *forward*：AGCTGTGATTTGCTGGGAGT  *reverse*： GACCAGATCCACTCTTGCCC |
| OT5 | *forward*：GTCCGCTGTCTTAAAGAGCCT  *reverse*： GGCGAGAGACCTCACTGAAT |
| OT6 | *forward*：CCCTGTCACCAAGTCACCTC  *reverse*： GATGCACCGTTTGAACTCGC |
| OT7 | *Forward：*CCAAGGAGTAGAACCCTGGC  *reverse*： TGCATTGAGAAAGCTCCCGT |
| OT8 | *forward*：GGGATTAGTGGAACTATGGTAAAGA  *reverse*： TTCGCCCTCTCCTTTTGTCA |
| OT9 | *forward*：CAAGAGGAACAGGCCCCATT  *reverse*： TGGGCCTTCAGGATGGTTTC |
| OT10 | *forward*：TGGGCTCCTATTTTCCTTACTGG  *reverse*：TGGAGCAAGGATGTGGAGTT |
| OT11 | *forward*：CCAGGAGATCTGAGTCCCCA  *reverse*： CTCTCAGGACCCACTACCGA |
