# Supplementary figures and images for: CRISPR/Cas9 facilitates investigation of neural circuit disease using human iPSCs: mechanism of epilepsy caused by an SCN1A loss-of-function mutation
Source: Transl Psychiatry. 2016 Jan 5;6(1):e703–. doi: 10.1038/tp.2015.203 (PMC5068877; doi:10.1038/tp.2015.203)

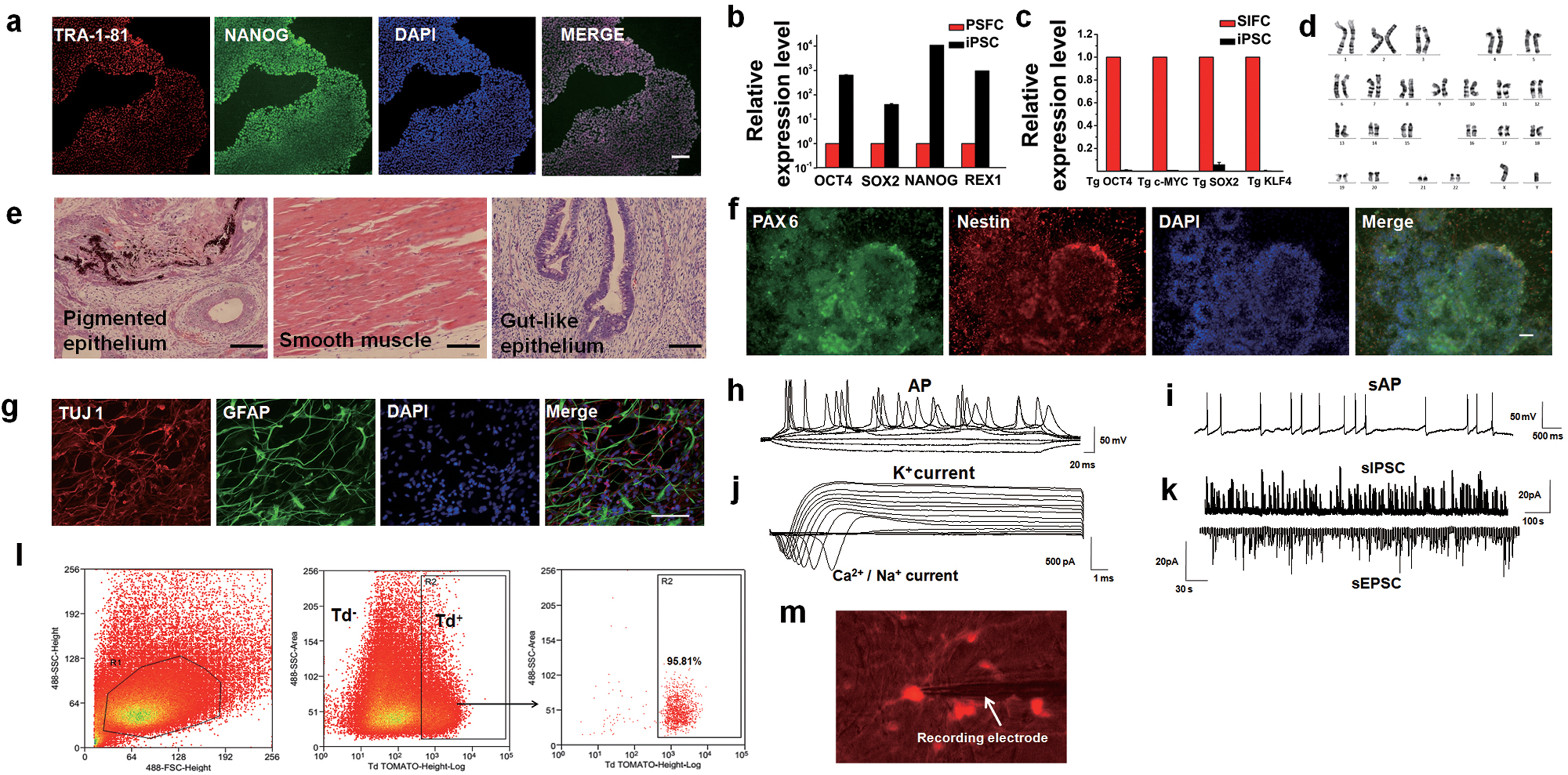

Supplement: Supplementary Figure 1 [file tp2015203x2.tif]

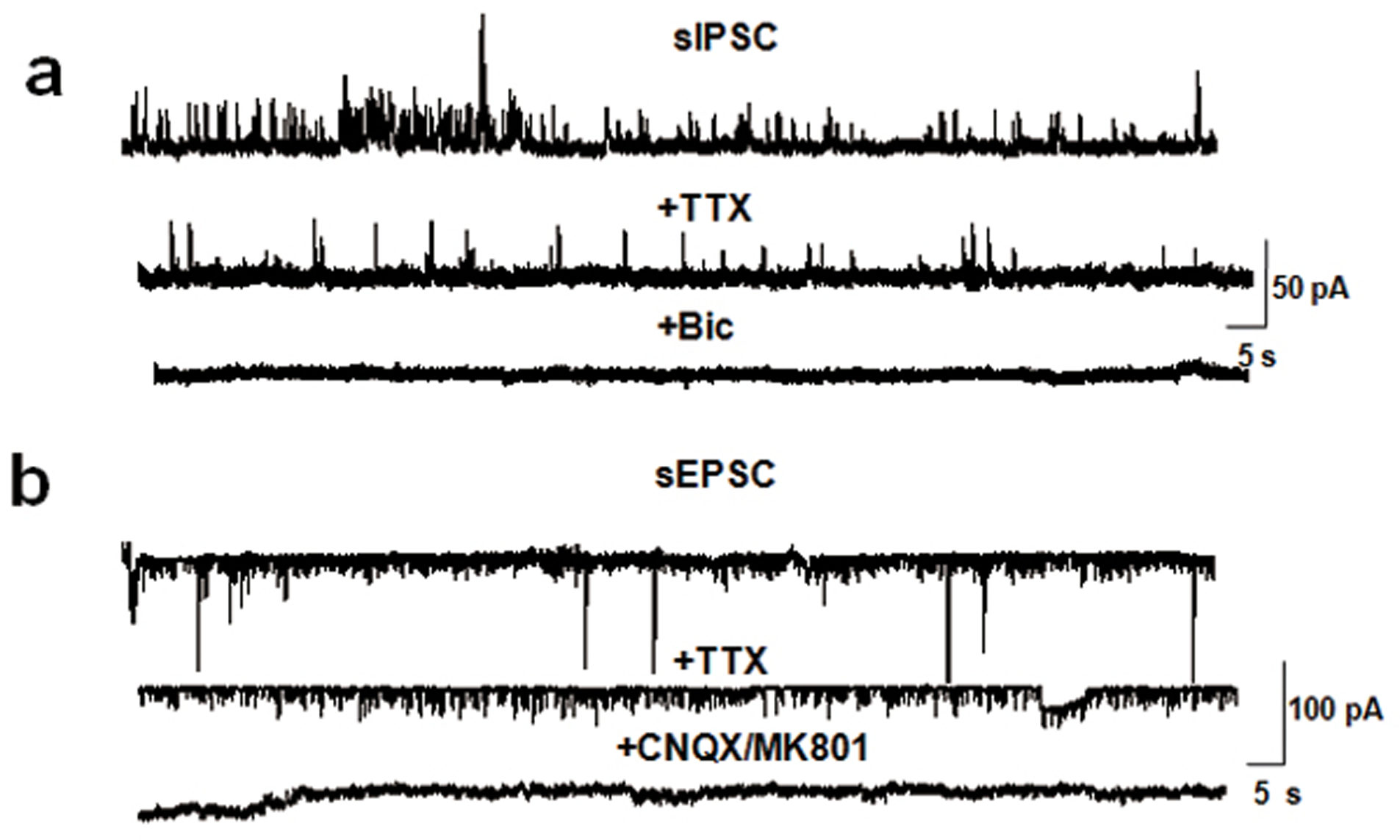

Supplement: Supplementary Figure 2 [file tp2015203x3.tif]
